# Integrating Growth Mindset with Functional-Cognitive Approaches: A Mixed-Methods Feasibility and Acceptability Study in Youth Residential Care

**DOI:** 10.3390/children13010148

**Published:** 2026-01-20

**Authors:** Miri Tal Saban, Sharon Zlotnik

**Affiliations:** 1 School of Occupational Therapy, The Hebrew University of Jerusalem, Jerusalem 9124001, Israel; miri.tal-saban@mail.huji.ac.il; 2 Occupational Therapy Department, Zefat Academic College, Zefat 1320611, Israel

**Keywords:** growth mindset, youth residential care, staff training, functional cognitive approaches, emotional regulation, psychosocial adjustment

## Abstract

**Highlights:**

**What are the main findings?**

- Residential staff provided strong emotional support but reported limited confidence in teaching functional and metacognitive skills needed for everyday autonomy.
- A pilot staff-based implementation of the integrated protocol was found feasible and acceptable, supporting the translation of motivational principles into adolescents’ daily functional strategy use.

**What are the implications of the main findings?**

- Implementing this protocol in residential care settings may inform staff-focused practices aimed at supporting adolescents’ functional autonomy and transition planning. Further evaluation is required to determine its impact on long-term outcomes.
- The feasibility of a pilot staff-based implementation offers a preliminary practical framework for staff-focused interventions in residential care settings.

**Abstract:**

**Background:** Adolescents in residential care frequently face functional challenges, yet few interventions integrate functional-cognitive models with motivational theories to support their daily function. **Methods:** This mixed-methods feasibility study is an innovative conceptual integration that links motivational and metacognitive approaches with growth-mindset principles to address both beliefs about the ability to change and functional performance. Quantitative data were collected from staff (n = 44), alumnae (n = 5), and current residents (n = 3), assessing mindset and functional-skill confidence among three focus groups (n = 16). The qualitative insights highlighted the motivational processes, strategy use, and barriers each group faced. **Results:** The findings informed the development of structured intervention psychoeducation protocol to facilitate goal-setting and reflective questioning. The feasibility and acceptability were tested by a pilot study among five staff members. Before implementation, staff demonstrated lower confidence in promoting daily autonomy and providing strategy-focused feedback. Alumnae and residents reported high emotional support, yet persistent gaps in functional independence. **Conclusions:** The pilot findings may inform the development of structured staff practices for delivering functional guidance, pending further evaluation. This study offers a novel conceptual contribution by positioning growth mindset as an active mechanism that supports functional-cognitive processes to enhance autonomy among adolescents in residential care settings.

## 1. Introduction

Mindset encompasses individuals’ assumptions, beliefs, and perceptions that determine how they interpret situations and respond to success and failure. Refs. [1,2] expanded this concept by distinguishing between individuals with a fixed mindset, who may fear challenges and view effort as evidence of inadequate innate ability, and those with a growth mindset, who believe abilities can be developed through effort, leading to curiosity, persistence, and willingness to learn from mistakes. A fixed mindset is associated with lower motivation and higher stress, whereas a growth mindset promotes engagement in learning, resilience to setbacks, and continued effort when faced with difficulties [2,3]. Mindsets exist on a continuum and are typically measured using self-report questionnaires that assess agreement with statements about the malleability versus fixedness of traits [4,5]. Research has linked a growth mindset to greater participation in daily activities and more effective self-management among adolescents [5,6,7,8].

During adolescence, the developmental period between ages 10 and 19 years, characterized by significant physical, cognitive, and psychosocial adjustments [9], social environmental factors—particularly feedback from authority figures and peer interactions—significantly shape mindset development [10]. Feedback focusing on effort promotes a growth mindset and better academic performance, whereas praise directed at intelligence fosters a fixed mindset and challenge avoidance [11]. Teachers who encourage a growth mindset enhance student classroom experiences and engagement in success-promoting behaviors [12]; however, peer comparisons and social status concerns can reinforce fixed beliefs about ability, especially during the identity-formation years of adolescence.

Nevertheless, the existing mindset research has focused predominantly on mainstream populations, creating a significant knowledge gap regarding at-risk adolescents. Specifically, although youth exposed to trauma and poverty tend to develop more fixed mindsets, compounding their achievement and mental health challenges [13], targeted mindset interventions for this vulnerable population remain largely unexplored. Some interventions can reduce anxiety symptoms and increase learning engagement among trauma-exposed students [14]; still, systematic approaches to mindset development designed specifically for at-risk youth remain critically needed.

Educational interventions to develop growth mindsets commonly teach participants the principle of neuroplasticity, the brain’s ability to form new neural connections through practice and learning. This understanding is typically conveyed through metaphors such as, “The brain is like a muscle; it gets stronger the more you use it,” to help learners internalize the concept [15,16]. Although most documented interventions focus exclusively on academic, emotional regulation, and social outcomes within educational settings, emerging research has suggested that mindsets are context-dependent; they vary across social, academic, and daily functioning domains based on individual experience [17,18]. This implication raises the critical question of how to promote growth mindset in daily functional abilities beyond academic contexts.

Evidence-based, task-oriented approaches offer valuable frameworks to address this gap. The cognitive orientation to daily occupational performance (CO-OP) approach uses guided discovery to help clients achieve functional goals by identifying cognitive strategies for daily living challenges [19]. Through structured questioning, clients select meaningful goals, develop action plans, discover problem-solving strategies, and reflectively examine their effectiveness [20]. Similarly, Toglia et al.’s [21,22] multicontext approach emphasizes skills transfer and generalization across contexts through structured mediation and reflection. Both approaches inherently and implicitly support growth mindset principles by encouraging personal goal setting, building self-efficacy through gradual achievement, and promoting metacognitive awareness of strategies and performance.

Ryan and Deci’s [23] self-determination theory (SDT) provides a motivational framework that complements growth mindset theory by explaining how environmental conditions foster or hinder intrinsic motivation and psychological growth. The SDT posits that optimal development occurs when the basic psychological needs for autonomy, competence, and relatedness are supported. In residential care settings, at-risk adolescents’ histories of control, dependency, and disrupted attachment frequently compromise these needs. Therefore, interventions that encourage adolescents to set personal meaningful goals, reflect on their progress, and experience success in manageable daily tasks can restore their sense of autonomy and competence [24,25].

When the facility staff models autonomy-supportive communication, such as providing choice, acknowledging emotions, or emphasizing effort over outcome, they help promote self-regulated motivation consistent with a growth mindset [5].

The Youth Custody Authority (YCA) serves adolescents whom courts have referred to due to delinquency, self-risk, and social danger under the Youth Judgment, Punishment, and Treatment Methods Law of 1971 [26]. These young people represent the most challenging cases, typically having exhausted treatment attempts in other settings. Recent decades have produced an increasingly vulnerable population with four times higher rates of emotional-behavioral problems compared to peers, extreme motivation deficits, and minimal social and family support [27]. The residential care institutions operate under systemic overload, high staff turnover, and limited formal training for direct care workers. In the face of these challenges, the YCA’s 24/7 holistic environment presents a unique opportunity for intervention, offering protection from community risk factors while providing comprehensive educational-therapeutic programming [28].

Despite extensive research on growth mindset in educational contexts, how these principles can effectively promote daily functional skills among at-risk adolescents in residential care is little understood. With their focus on school settings, existing studies tend to neglect the functional-cognitive capacities adolescents require for autonomy and community participation. This gap highlights the need for theoretically grounded, context-specific interventions integrating growth mindset theory with occupational and functional frameworks.

Given the limited research on promoting a growth mindset focused on daily functional abilities within YCA settings and the lack of appropriate tools for this population, our study aims to develop and assess the feasibility of an intervention protocol to cultivate a growth mindset. This intervention specifically targets everyday functional abilities among adolescents in the YCA, addressing the identified gap between theoretical mindset beliefs and practical daily functioning skills necessary for successful community integration. The study extends growth mindset research by integrating functional-cognitive approaches within the YCA.

To address the gap between theoretical growth-mindset principles and their application to daily functional abilities in residential care, this study examined the perspectives of staff, alumnae, and current residents to inform the development of an intervention integrating growth mindset with functional-cognitive approaches.

**Figure 1:**
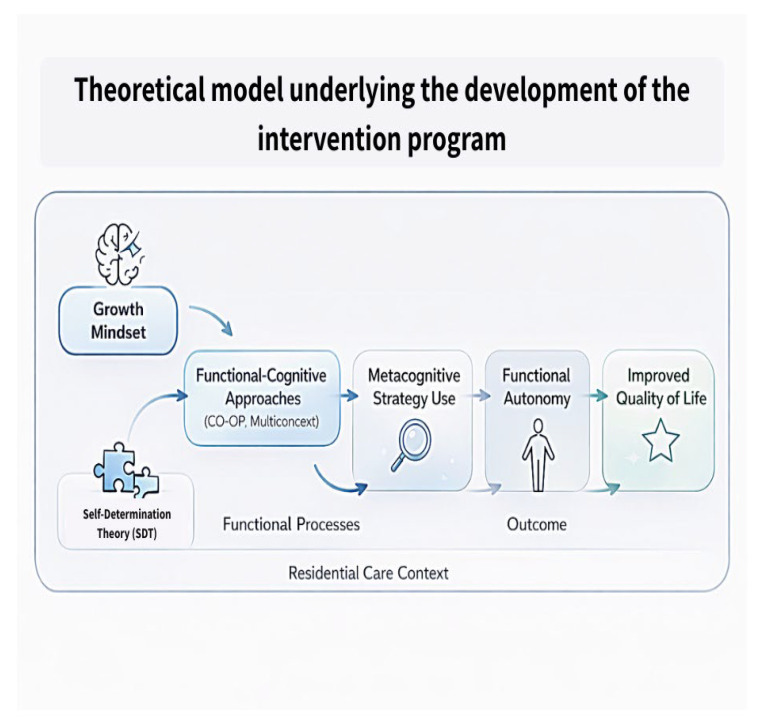
Theoretical model underlying the development of the intervention program.

As can be seen in Figure 1, the proposed theoretical model integrates Self-Determination Theory (SDT) with growth-mindset principles, with metacognitive functional cognition approaches (CO-OP; Multicontext) and autonomy development in residential care. Growth mindset fosters openness to learning and strategy use, which facilitates metacognitive problem-solving. These processes enhance functional cognition and everyday executive functioning, supporting independent living skills. The residential care environment serves both as the context of performance and as a feedback system that reinforces or constrains autonomy.

Accordingly, this study was designed as a feasibility and acceptability study. Rather than evaluating intervention effectiveness, the study aimed to examine whether an integrated growth mindset–functional-cognitive protocol could be implemented within the organizational constraints of residential care settings and whether it would be perceived as acceptable by staff.

The study asked: (a) how growth-mindset beliefs and functional-cognitive practices are currently expressed in the Youth Custody Authority (YCA); (b) what barriers and facilitators shape functional autonomy; and (c) whether such an integrated intervention is feasible and acceptable for use in residential care settings. These questions guided the design and feasibility assessment.

**Hypotheses:** (1) Staff will report strong relational abilities and belief in youth change capacity but lower confidence in teaching functional and organizational skills. (2) Current residents and alumnae will report high emotional support but persistent gaps in functional autonomy and independent-living skills. (3) An intervention integrating growth-mindset and functional-cognitive principles will be feasible and acceptable for staff to implement. (4) Quantitative and qualitative findings will converge in identifying the need for structured, strategy-focused approaches to promote functional autonomy.

## 2. Materials and Methods

### 2.1. Study Design

This feasibility and acceptability study used a mixed-methods design to identify barriers and facilitators for promoting growth mindsets among adolescents in residential care settings and develop an evidence-based intervention protocol. In this study, feasibility refers to the extent to which the intervention protocol could be implemented within the routine organizational constraints of residential care, including staff availability, workload, and crisis-driven interruptions. Acceptability refers to staff perceptions of the relevance, usefulness, and practicality of the protocol for everyday work with adolescents. During acute crisis situations, such as runaways or outbursts, staff appropriately prioritized immediate safety and stabilization, and the protocol was not consistently applied. This reflects real-world implementation constraints rather than protocol non-adherence. The mixed-methods design, integrating quantitative data on mindset perceptions with qualitative insights into daily practices, was chosen to capture measurable patterns and experiential perspectives and ensure an ecologically valid and context-responsive intervention.

**Figure 2:**
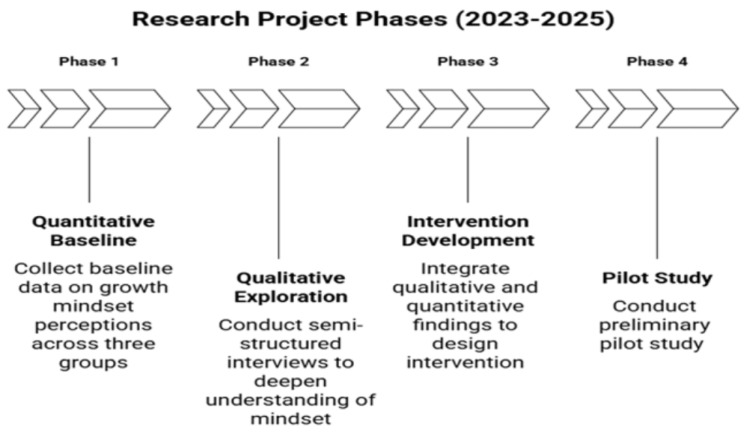
Research Project Phases.

We conducted research between 2023 and 2025 in four interconnected phases (illustrated in Figure 2). Phase 1 (quantitative) provided baseline data on growth mindset perceptions across three groups, which informed the development of semi-structured protocols for Phase 2 (qualitative). The Phase 1 quantitative findings were integrated with Phase 2 qualitative results to guide Phase 3 (intervention development), and Phase 4 (preliminary pilot study). This sequential approach enabled a comprehensive understanding of the phenomenon while grounding intervention components in empirical evidence from multiple perspectives.

### 2.2. Participants

#### Phases 1 and 2

A total of 52 participants, ranging in age from 16 to 55 years, were recruited across three groups within residential care settings: residential care staff, former residents or “alumnae,” and current residents.

**Residential Care Staff:** In Phase 1, 44 staff members representing two facilities (one for adolescent boys and one for adolescents girls) participated. This sample was predominantly women (73%); 39.5% were single, 34.9% were in committed relationships, and 7% were married. They included educational counselors (*n* = 23), social workers (*n* = 7), program coordinators (*n* = 6), administrators (*n* = 4), emotional health practitioners (n = 3), and administrative personnel (*n* = 1). These participants ranged in age from 24 to 55 years (*M* = 35.2 years, *SD* = 8.4) and varied in educational background (47% held bachelor’s degrees, 21% completed secondary education, and 12% held advanced degrees) and professional experience (3 months to 15 years; most reported 1–3 years). Nine staff members (one male and 8 females) from the girls’ residential facility participated in Phase 2 (focus groups).

**Former Residents (Alumnae) of the Girls Facility:** In Phase 1, five young women who had previously resided at the girls’ residential facility (for 1–4 years) participated. They ranged in age from 18 to 24 years (*M* = 23.4 years, *SD* = 2.1). All had been living independently in the community for at least 6 months at the time of participation. Most (80%) were unmarried, had completed secondary schooling but no matriculation, and were employed in entry-level positions requiring minimal qualifications. Three of these participants also participated in Phase 2.

**Current Residents of the Girls’ Facility:** Three adolescents currently residing at the residential facility participated in Phase 1, and four participated in Phase 2. They ranged in age from 16 to 18 years (*M* = 17.1 years, *SD* = 0.8) and had been in residential care for 3 months to 3 years.

### 2.3. Focus Group Participants

The qualitative phase of this study comprised three focus groups conducted within a single residential facility for at-risk adolescent girls. Ultimately, we proceeded with the girls’ residential facility only, as the boys’ facility was unable to organize participation in the study, and we therefore selected the site that was able to commit to the research. Sixteen participants represented three stakeholder groups from the facility (nine staff members, four current residents, and three program alumnae).

### 2.4. Inclusion and Exclusion Criteria

Inclusion criteria for participants among the staff were (a) direct client contact, (b) minimum 3 months of facility experience, and (c) willingness to contribute professional insights. For alumnae, the criteria required (a) completion of at least 1 year of residential care, (b) current emotional stability, (c) ability to reflect on transition experiences, and (d) voluntary participation. Criteria for current residents were (a) emotional stability, as assessed by the clinical team, (b) demonstrated ability to engage in reflective dialogue, (c) minimum 3–month facility residence, and (d) informed consent from the participant and their legal guardian.

Exclusion criteria across all groups were (a) acute mental health instability, (b) history of severe trauma responses to retrospective discussions, (c) clinical team assessment that participation might compromise therapeutic progress, (d) current personal crisis, and (e) inability to provide informed consent.

### 2.5. Recruitment and Ethical Considerations

In coordination with the research team, facility administrators helped recruit participants. To minimize coercion, alumnae were contacted exclusively by research assistants rather than facility staff, and current residents required additional consent (i.e., from legal guardians or facility administrators). All participants received comprehensive information about the study’s objectives, procedures, rights, and confidentiality protections before providing written informed consent.

The study was conducted in accordance with the approved by the Human Research Ethics Committee of The Hebrew University of Jerusalem, Israel [#5032023], (date of approval 5 March 2023) and the Ethics Committee of the Ministry of Welfare Jerusalem, Israel (date of approval 4 May 2023). The study was prospectively registered on Clini-calTrials.gov (#NCT06824012). We maintained participant anonymity through unique identifiers, with all identifying information removed from transcripts, and stored data on secure, access-restricted servers. The research team was available during all data collection sessions, and group facilitators had been trained to recognize and appropriately respond to signs of participant distress. Participation in the focus groups was entirely voluntary, and adolescents were not required to share personal experiences.

Participants included residential care staff, current residents, and program alumnae. The primary unit of intervention and analysis in this feasibility study was the residential care staff. Youth data were collected to contextualize needs and implementation targets, rather than to evaluate youth outcomes.

### 2.6. Measures

#### 2.6.1. Quantitative Instruments (Phase 1)

**Demographic Questionnaire.** A research-developed questionnaire collected information on age, gender, education level, professional role (staff only), and residential care history (current residents and alumnae).

**Growth Mindset Facilitation Self-Efficacy questionnaire:** This questionnaire was specifically designed for this study, based on growth mindset theory [3] and recent conceptual refinements [5]. The questionnaire assesses three domains: (a) beliefs about personal change capacity (eight items), (b) general self-efficacy in daily functioning domains, including self-management and community integration (12 items), and (c) confidence in managing specific functional challenges (10 items). Items are rated on a 10-point Likert scale ranging from 1 (strongly disagree) to 10 (strongly agree), with higher scores reflecting stronger growth mindset facilitation self-efficacy orientations. Pilot testing of the questionnaire with 15 participants from similar populations yielded acceptable internal consistency (Cronbach’s α = 0.84). Item-level descriptive analyses revealed moderate to high mean scores with adequate variability (means approximately 6.0–8.2), suggesting no pronounced floor or ceiling effects. We used this questionnaire among the residential staff twice, in phase 1 (baseline) and phase 4 (Pilot), and once among the alumnae and female current residents in phase 2 (focus groups).

#### 2.6.2. Qualitative Instruments (Phase 2)

**Focus Group Protocol**. We developed semi-structured interview guides for each of the three participant groups based on growth mindset theory, CO-OP, the multicontext approach, and SDT (Supplementary File S1). Themes addressed in the interviews included self-efficacy beliefs, goal orientation, experiences with success and failure, coping strategies, sense of belonging, autonomy development, and change processes. The pilot tested and refined the protocols based on feedback from clinical experts and initial participant responses.

### 2.7. Procedure

#### 2.7.1. Phase 1: Quantitative Data Collection

Participants completed the questionnaires via the Qualtrics survey platform.

#### 2.7.2. Phase 2: Qualitative Data Collection

Following the quantitative analysis across all facilities, we conducted focus groups only at the girls’ residential facility. The residential staff focus groups (*n* = 9 participants) and the current resident focus groups (*n* = 4 participants) were held in the YCA at special times face-to-face. The alumnae focus groups (*n* = 3 participants) were conducted via videoconference to accommodate geographic dispersion and reduce facility-related anxiety (see Table 1 for more details). Each focus group lasted 60 to 90 min and was audio recorded with participant consent.

An important feasibility finding concerned the need to adapt the original multi-component protocol to a dyadic implementation model in response to contextual constraints within the residential care setting, including staff availability, shift structure, and crisis-driven priorities. This adaptation indicates that the dyadic configuration constituted the feasible implementation format examined in the preliminary pilot phase.

#### 2.7.3. Phase 3: Intervention Development

Using data triangulation, we integrated Phase 1 and Phase 2 quantitative and qualitative findings to identify key intervention targets. The resulting protocol incorporated group sessions, individual mentoring, and peer-facilitated activities based on Growth mindset, SDT, CO-OP and Multicontext approaches, emotional regulation strategies, and functional reflection. It targeted enhanced emotional, behavioral, and sensory self-regulation.

Although the intervention was originally designed as a multi-component protocol including group sessions and peer-facilitated activities, implementation constraints led to a shift toward an individualized dyadic format during the pilot phase. This shift was therefore treated as a feasibility-driven design adaptation rather than a protocol deviation, and the pilot findings reflect the feasibility of the dyadic configuration rather than the full multi-component protocol as originally designed. While the delivery format shifted to a dyadic model, the pilot preserved the core intervention principles, including guided discovery, goal anchoring, reflective dialogue, and functional task analysis. Group-based and peer-facilitated components were not implemented during the pilot phase.

#### 2.7.4. Phase 4: Preliminary Pilot Study

The intervention protocol was implemented with five volunteer staff members over an eight-week period. The pilot phase was embedded within routine residential care practice rather than delivered as a series of standardized sessions. Data collected through descriptively structured reflection logs and post-intervention interviews were used to examine whether and how core intervention principles were applied during routine practice, as well as to assess the protocol’s feasibility and preliminary acceptability.

### 2.8. Data Analysis

#### Quantitative Analysis

We calculated descriptive statistics, including means, standard deviations, and frequency distributions, for all questionnaire data. Given the small sample sizes and cross-sectional design focused on needs assessment rather than hypothesis testing, no inferential hypothesis-testing analyses (e.g., group comparisons or predictive models) were conducted. Exploratory Spearman correlation coefficients were calculated to examine associations between self-efficacy domains in order to identify patterns relevant to needs assessment and intervention development [29].

### 2.9. Qualitative Analysis

The transcription of all audio recordings verbatim and analyzed was conducted using a qualitative content analysis method with a directed approach [30]. The focus-group data were processed using transcript-based analysis per [31] recommendations. Specifically, the focus group recordings were transcribed and the contents analyzed to identify narratives grounded primarily in the growth mindset theory, as well as the CO-OP, multicontext approach, and SDT. First, the process involved introducing the focus group content, creating initial codes, searching for themes, and checking the codes for correspondence with the themes. Themes were identified within and across participant groups, with particular attention to convergent and divergent between-group perspectives. Two independent researchers conducted the initial coding, coded the elicited quotes, and resolved disagreements through consultation with a third researcher and discussion until they reached consensus.

### 2.10. Integration of Findings

Quantitative and qualitative data were integrated through data triangulation [32], comparing numerical patterns with thematic findings to identify areas of convergence and divergence. This integrated analysis informed selection, structure, and sequencing of the intervention component, as well as the implementation strategies, ensuring the final protocol addressed empirically identified needs while remaining feasible within residential care contexts.

Generative artificial intelligence tools were used for general language editing and refinement and to assist with the visual design of conceptual figures. No generative AI was used to generate study data, analyze results, or interpret findings. All scientific content and conclusions are solely the responsibility of the authors.

## 3. Results

Reported changes refer to staff practices and perceptions; references to youth interactions are presented to illustrate implementation contexts rather than to indicate youth-level outcomes.

### 3.1. Phase 1: Quantitative Findings

#### 3.1.1. Residential Staff Perceptions and Growth Mindset

**Baseline Assessment**. Using the Growth Mindset Facilitation Self-Efficacy questionnaire, the baseline survey included 44 staff members from two residential facilities (one serving adolescent boys and one serving adolescent girls). Relational skills received the highest ratings: trust-building (*M* = 9.11, *SD* = 1.23), peer-support facilitation (*M* = 8.84, *SD* = 1.30), and maintaining belief in youth potential (*M* = 9.06, *SD* = 1.60). These results aligned with the SDT emphasis on relatedness as a core psychological need [23].

In contrast, preparing youth for functional independence, where higher scores indicate greater confidence, received lower ratings (*M* = 6.4, *SD* = 2.88). The staff reported their lowest confidence in teaching organizational skills (*M* = 5.55, *SD* = 2.81) and in promoting daily autonomy (*M* = 5.91, *SD =* 2.85). A strong positive correlation (*r* = 0.76, *p* < 0.001) was observed between perceived ability to foster independence and capacity to motivate youth, indicating a meaningful relationship between these domains in this sample. Descriptive statistics indicated slightly higher mean scores among male staff compared to female staff in relational competence (men: *M* = 8.73, *SD* = 0.50; women: *M* = 8.41, *SD* = 1.62) and functional skill development (men: *M* = 7.24, *SD* = 1.39; women: *M* = 6.90, *SD* = 2.08); however, no inferential gender comparisons were conducted.

**Reassessment was** conducted in phase 4 to characterize the population in which to conduct the preliminary pilot study. Specifically, collecting data from 11 staff members at the girls’ facility (*M*_age_ = 44.0 years, *SD* = 14.75). Most (81.8%) were educational counselors, and the remainder were social workers. Workforce turnover was evident: 72.7% had 2 years or less of tenure, and only 9.1% had more than 5 years.

Upon reassessment, the staff’s mean self-efficacy scores indicated moderate competence: They felt relatively confident teaching coping strategies (*M* = 5.91, *SD* = 1.22), facilitating daily problem-solving (*M* = 5.45, *SD* = 0.93), and encouraging growth-mindset thinking (*M* = 5.45, *SD* = 1.03). These results aligned with the multicontext approach, which emphasizes strategy training and flexible application (Toglia, 2011) [33]. However, the staff reported lower competence in identifying sensory processing needs (*M* = 4.27, *SD* = 1.01) and using growth mindset language (*M* = 4.36, *SD* = 1.74). Notably high variability was observed (*SD* > 1.5) in group facilitation and motivational strategies. This result indicated uneven professional readiness, likely to reflect the high proportion of less experienced staff.

#### 3.1.2. Alumnae Experiences

The five female alumnae evaluated facility support as extremely high, with maximum ratings (10/10) for personal safety, staff assistance, community belonging, and crisis support. They emphasized enduring positive staff relationships extending beyond their residency. Despite these strengths, the alumnae highlighted gaps in autonomy preparation. Ratings for independent self-management (*M* = 6.8, *SD* = 2.17), functional autonomy (*M* = 7.0, *SD* = 3.74), and daily organization (*M* = 7.2, *SD* = 2.68) were consistently lower. These findings mirror staff-identified gaps in preparation for functional independence, suggesting systemic challenges.

#### 3.1.3. Current Residents

All three current residents reported very high satisfaction with staff support and trust (e.g., on a 1–10 scale, the means for trust in staff and perceived availability were 9.00–10.00, *SD* 0.00–1.73) and a strong sense of safety. In contrast, ratings related to autonomy (*M* = 5.33, *SD* = 0.58) and peer relationships (*M* = 6.67, *SD* = 2.89) were more variable, underscoring diverse individual experiences. This variability supports the CO-OP model’s emphasis on individualized goal setting and strategy use.

### 3.2. Phase 2: Qualitative Findings

#### 3.2.1. Thematic Structure

The thematic content analysis yielded a hierarchical framework comprising two superordinate categories and seven principal themes with constituent subthemes. The first category, Facilitating Factors, encompasses strengths and existing practices within the facility that support the development of a growth mindset and functional autonomy. The second category, Implementation Challenges and Systemic Concerns, identifies areas requiring targeted intervention and structural enhancement. Table 2 provides a detailed taxonomy of all themes and subthemes identified in the analysis.

#### 3.2.2. Category I: Facilitating Factors

Theme 1: Growth Mindset Integration and Therapeutic Language. The focus groups revealed that staff intentionally used growth-mindset language to reinforce competence and belief in change. As one staff member expressed, “I think they are able to change thinking patterns” (S-3). The alumnae corroborated this observation, describing transformative shifts in self-perception. One reflected,

I never believed in myself. I knew I could, but not enough. And they changed my perception, and it allowed me to understand that there is someone here to whom you can say what you want, and they will be attentive and be with you. (A-1)

Current residents also articulated personal agency in their descriptions of daily life at the facility. As one youth noted, “Everything here is our choice. I can take the phone, but there will be consequences. The question is whether I choose correctly or not” (Y-2). These examples illustrate both autonomy and competence support, consistent with SDT [23].

The growth-mindset discourse extended beyond staff interactions to peer relationships as well. One youth described the impact of peer support: “She believes in me, to this day, that I’ll succeed, believes I’ll get a driver’s license. Believes in many things, in things I don’t believe in. It’s empowering. It’s contagious, that belief” (Y-3). This peer modeling suggests successful internalization of growth mindset principles within the broader facility culture.

**Theme 2: Collaborative Goal setting and Youth Agency.** Goal setting emerged as a youth-led and a staff-supported process, aligning with CO-OP principles of guided discovery [34]. The staff emphasized that the youth could formulate goals independently. One staff member stated, “*Of course they can set goals for themselves. They will set goals and say what they want*” (S-4). The youth participants confirmed this autonomy. One noted, “*It’s also what appears in my personal plan, to help new girls acclimate*” (Y-1).

At the same time, the staff deliberately introduced challenges to expand comfort zones and generate mastery experiences. One staff member explained,

“We arrive on Friday mornings and pull them from their beds, the only time they can rest without someone bothering them, and they manage to mobilize and get up. Especially the girls who go out and leave their comfort zones, they reveal themselves at their peak”.

(S-7)

This balance between youth-directed and staff-initiated goals appeared to support autonomy and competence development.

However, the youths’ perspectives revealed that motivation remained contingent on internal readiness. One resident explained, “To achieve goals, first I need my motivation. That’s it. It will help me if I start doing things, not just talking about things in the air. The staff can’t help with anything. Not that I can’t, I don’t have the energy”. (Y-4).

This quotation suggests that although goal-setting structures were in place, translating motivation into sustained action remained a complex challenge.

**Theme 3: Environmental Structure and Regulation.** Routine and predictability emerged as critical protective factors across all participant groups. One alumna described the regulatory function of structured schedules: “*There was always a schedule. There wasn’t a moment when you had, ‘Ah okay, what am I going to do now.’ You were always busy, and for me personally it helped a lot” (A-2). She elaborated on the value of clear boundaries: “There were very clear rules, red lines that you don’t cross, no matter what happens. And they maintained those rules*” (A-2).

This structured environment appeared to serve multiple regulatory functions. It maintained consistent sleep–wake cycles, provided ongoing occupational engagement, and reduced the cognitive load associated with constant decision-making. These findings align with the multicontext approach’s emphasis on environmental supports to scaffold performance and regulation [33].

Further, the staff recognized this organizing principle, although some questioned whether the high level of structure adequately prepared youth for less structured community living. One staff member wondered aloud whether the residents had developed sufficient skills for environments where they would need to create their own structure (S-4). This tension between providing regulatory support and promoting independence became a recurring theme throughout the analysis.

**Theme 4: Relationship-Based Engagement.** Relationships with staff emerged as central motivators for behavior change. One alumna candidly admitted, “*I wouldn’t do it, so as not to disappoint the staff, not to destroy everything I built with the staff until now*” (A-3). This extrinsic motivation, rooted in relationship preservation, appeared to function as a developmental bridge toward more internalized forms of motivation.

The staff members highlighted nonjudgmental attitudes as essential to building the trust necessary for change. One explained, “*When they feel lack of judgment from the staff toward them, that’s what opens a direct channel to their heart*” (S-5). Another elaborated on this dynamic:

*“The more she feels safe and the more she feels seen and heard here, the more comfortable she’ll feel changing herself. Recognizing that sometimes her choices aren’t good for her. She won’t be defensive, thinking we are wrong, that we want bad for her”.*

*(S-8)*

These relational dynamics illustrate the role of relatedness in promoting internalized motivation, as described in SDT [33]. Importantly, peer relationships also played a significant role. The youth described how their relationships with one another reinforced positive change and provided mutual support during difficult moments. One noted that the belief and encouragement from peers felt “*contagious*” and empowering (Y-3).

#### 3.2.3. Category II: Implementation Challenges and Systemic Concerns

**Theme 5: Feedback Practices and Reflective Learning.** Our analysis revealed that the facility staff’s feedback mechanisms emphasized outcomes rather than reflective strategy-building. One staff member described her approach as follows:

*“I give meaning through constant reflection. In the morning, I give a word about whether she met expectations or didn’t meet them, to reflect on it to her and show her. Also, when they improve, to show that too. Look how you couldn’t get up for a week, now get up for 2 days, today you already managed. Things like that. It seeps in, they begin to understand how to manage a relationship with themselves”.*

*(S-2)*

Although this approach was supportive and well-intentioned, it focused predominantly on behavioral outcomes (e.g., successfully waking up) rather than on the strategies used to achieve them. Contemporary theoretical frameworks highlight the importance of formative feedback that targets processes and promotes strategy transfer [35].

Questions such as “How did you manage to wake up?” or “What did you do to get up on time?” were notably absent from the feedback practices participants described.

The alumnae and the current residents expressed a need for more process-oriented guidance. As one alumna reflected on staff interactions,

*“You need to learn how to approach a girl who is currently in a frustrating state. In the end, we all went there for a certain reason. It’s not a hotel or a camp. We weren’t there voluntarily. Behind every girl stands a story she carries with herself, and she needs that thing”.*

*(A-1)*

This comment suggests that understanding the “how” of successful coping, not just the “what” of outcomes, was critically important to residents.

**Theme 6: Transition to Independent Living.** Despite strong relational outcomes and positive experiences within the facility, both staff and alumnae emphasized significant difficulties in preparing for independent living. The staff noted that practical tasks, such as renting an apartment or interacting with external services, often triggered considerable anxiety. One staff member described,

*“The difficulty is very, very big, the panic very, very big, that they have about talking to people not connected to the facility. That is, if they need to talk to customer service, to talk to the doctor. Many times, even when they want to do it, there’s some guidance during and we sit next to them to do it. Very quickly it’s like, you take it, you talk”.*

*(S-6)*

The alumnae confirmed these challenges from their lived experience. One explained, “Not every girl who leaves here leaves with a great sense of success. There will be a gap between how she finishes here and what happens at home” (A-3). Another provided a poignant description of post discharge adjustment:

*“It was like residence, residence, like that. And suddenly, when I went out to the world in real life, before the army, I got such a slap. I got a slap, and now when I was discharged from the army, even more of a slap. But I think if there was more follow-up, for me personally, it would have helped. A phone call, what’s happening, how are you, everything okay, are you managing, do you need anything, like that. Not expecting every day. Yes, for a few months we were still in touch, until slowly it disconnected. I’m currently experiencing this, and it’s very hard. Yes, I need help now, let’s say”.*

*(A-2)*

These findings underscore the need for structured transition planning with explicit transfer strategies, as emphasized in the CO-OP [32] and multicontext approaches [22]. The alumna’s metaphor of a “slap” powerfully conveys the jarring disconnect between the highly structured, supportive facility environment and the less predictable, less supported community context.

**Theme 7: Staff Training and Professional Development.** The alumnae were particularly vocal about perceived gaps in staff preparation, given the complexity of the residents’ trauma histories and behavioral needs. One alumna emphatically stated, *“You can’t just enter to work here and learn as you go. Because, ultimately, we are girls who have many complexities and many emotional things that a stranger won’t understand*” (A-1). Another emphasized the professional nature of the work:

*“Maybe they just need to enable the staff to do their work in a more correct and professional way. This should be the most professional in the world, to work with at-risk girls. It’s not that anyone can just come. You need people who know how to approach”.*

*(A-3)*

The current residents echoed these concerns, calling for more individualized approaches.

*“When I tell someone to back off because I’m angry, I mean it. So why, when people still come near me, does it make everything worse, like it throws me into this dark place. There was a point where I just lost it, like really lost it. I flipped half of the place, smashed the storage room, …. broke cups everywhere. They tried to stop me, and I kept saying, don’t touch me. […] It’s hard when someone comes close to me. I can totally lose control, like go completely wild.”*

*(Y-2)*

The residents provided specific behavioral examples of approaches that felt counterproductive or retraumatizing. One described, “*Don’t yell in my ear and turn on the light. I have hands. Tell me, bring the hands, lift my hands, lift me from the bed. Don’t need to yell, don’t need to turn on the light, don’t need to darken my whole morning*. (Y-4)

These accounts highlight the need for structured training in trauma-informed care, particularly for newer or younger staff members who may lack experience with the complex trauma histories and behavioral dysregulation common in this adolescent population. These findings align with research demonstrating that paraprofessional staff can serve as effective change agents when they receive structured training, clinical supervision, and supportive organizational environments [36].

### 3.3. Phase 3–4. Intervention Development and Implementation

The intervention protocol was directly informed by the qualitative findings. Facilitating factors (Themes 1–4) guided the preservation of relational, structural, and growth-mindset mechanisms, while implementation challenges (Themes 5–7) explicitly shaped the focus on metacognitive strategy instruction, preparation for functional independence, and structured staff training. Triangulation across stakeholder perspectives revealed strong relational foundations alongside critical gaps in metacognitive strategy instruction, functional independence preparation, and systematic staff training. These findings informed the development of a CO-OP-based intervention protocol to bridge the gap between growth mindset discourse and functional performance, while remaining feasible within the resource constraints and organizational realities of residential youth protection settings. The protocol prioritizes explicit strategy instruction, graduated transfer planning, and accessible training materials for staff with varying levels of formal preparation. The following sections describe the protocol and preliminary feasibility findings.

The intervention protocol was developed through a mixed methods process that integrated questionnaire data and focus group findings. Drawing on functional cognition, guided discovery (CO-OP), and metacognitive principles, the protocol aimed to enhance the staff’s capacity to promote a growth mindset and functional independence while addressing operational constraints.

The intervention comprised three sequential phases implemented between 2023 and 2025. The first phase consisted of foundational psychoeducational training delivered through four structured sessions (two in-person, two online) that introduced core concepts of growth mindset theory and its application to daily functioning. Content included executive functioning, goal formulation, organizational strategies, sensory-behavioral regulation, and reflective communication techniques. The staff practiced distinguishing between fixed and growth-mindset language patterns and applying formative feedback that emphasizes effort, strategy deployment, and learning from error.

The second phase focused on guided practice through individual and small group guiding sessions lasting approximately 2.5 h each. Drawing on guided discovery methodology, facilitators supported counselors in translating theoretical knowledge into practical application, specifically in formulating achievable functional goals with youth, monitoring progress, and fostering metacognitive reflection. During this phase, five to eight consecutive supervision meetings were conducted in triads, strengthening staff competency in realistic goal setting, promoting adolescent self-reflection, and implementing structured questioning techniques during naturalistic interactions.

The third phase involved field implementation adapted to address contextual constraints identified during pilot testing at the custody facility. Rather than full-group facilitation, as originally designed, the protocol shifted to an individualized dyadic model, pairing each counselor with one adolescent for goal-mindset attainment. Counselors conducted regular meetings during evening shifts, focusing on formulating and tracking proximal functional goals linked to objectives that include maintaining family relationships, educational attainment and employment readiness.

Periodic reflective consultations with the lead facilitator addressed implementation of strategies to sustain motivation and self-efficacy, while linking micro-level behavioral achievements to broader adaptive beliefs about the ability to change and personal competence.

The protocol operated on six core principles: guided discovery through questioning, rather than direct instruction; goal-setting anchored in meaningful, achievable functional objectives; connecting daily task successes to adaptive self-beliefs; promoting autonomy through structured routines and emotional regulation awareness; staff modeling of growth-mindset language in everyday communication; and establishing relational safety and emotional containment as prerequisites for learning. These principles were operationalized through structured dialogue protocols, goal-monitoring frameworks, and reflective practice guidelines tailored to the residential care context.

During implementation, counselors used brief reflective prompts embedded in everyday interactions to support metacognitive reflection and motivation. For example, questions such as “What strategy helped you manage this situation?” and “What did you do differently when it worked?” were used to promote guided discovery and strategy awareness (CO-OP). Prompts such as “What helped you keep trying when it was difficult?” reflected growth-mindset principles, while questions including “What choice felt most under your control in this situation?” supported autonomy and competence, consistent with SDT. These prompts were applied flexibly rather than as a scripted tool, allowing adaptation to individual youth needs and contextual constraints.

During the implementation phase, counselors described personal challenges and insights that shaped their work with the adolescents, reflecting the process of learning to set personal goals and forming a meaningful connection with the adolescents. This reflected the transition from an unsure and dissatisfied counselor to perceiving oneself as a mentor. As one counselor noted (S-3): *“During the supervision process, I was preparing to leave for a long trip and wasn’t sure I would return. My goals focused on how to part from the girls in a meaningful way. As someone who grew up at risk myself, I wanted to think carefully about the message I could leave them with about building a life and work relationships and choosing to travel the world.” …….”In the end we shared a cooking and farewell meal, I also prepared a small personal gift for each girl something that reflected the message I wanted to pass on as a mentor.”*

The counselors also described ways in which the learning process enhanced their ability to guide and respond to the adolescents: One counselor (S-4) identified that his primary challenge was responding to the girls in a calm, deliberate manner, and he worried that his quick reactions might model unhelpful patterns for them. Through the protocol, he set a goal of serving as a positive role model by intentionally pausing and verbalizing his thinking before responding. He practiced this by stating explicitly when he needed a moment to consider his reaction, for example, when a girl spoke to him disrespectfully, he would say that he wished to pause the conversation and think about how to respond. Similarly, when he suspected a rule had been broken and the girl denied it, he used the strategy of explaining that he wanted to consult with the team before deciding how to proceed. He reported successful use of these techniques in several interactions, noting that they helped him feel more grounded and thoughtful. Although a later transition to night shifts made continued practice more difficult, he expressed increased confidence in his ability to model reflective and regulated behavior for the girls.

Across cases, counselors highlighted how the structured, collaborative approach helped them link long-term aspirations with short-term, manageable actions. As one counselor summarized, *“It became about helping them take a dream and turn it into something they can start doing tomorrow.”* These reflections demonstrate the counselors’ growing skill in applying the protocol’s principles and their success in enabling the girls to define purposeful goals and experience attainable progress

## 4. Discussion

This study examined the feasibility and acceptability of integrating growth mindset principles with functional-cognitive approaches in residential care as a preliminary step before evaluating the intervention effectiveness.

The convergence of quantitative and qualitative data reveals a theoretically significant and consistent pattern across all participant perspectives. The staff demonstrated strong foundational capabilities in building relationships and applying growth mindset language, evidenced by high self-efficacy ratings in trust-building and belief in youth potential. Alumnae testimonials describing transformative relational experiences that endured beyond facility departure corroborated these quantitative findings. However, substantial gaps emerged in translating these motivational strengths into structured, functional, daily life skills preparation, as reflected in notably lower self-efficacy ratings for organizational skills instruction and independence promotion.

This pattern suggests that although YCA staff intuitively grasp growth mindset principles at the relational level, they require systematic training to bridge theoretical understanding with functional skill application. The strong positive correlation between staff confidence in encouraging independence and facilitating youth motivation provides empirical support for integrating growth mindset principles with functional-cognitive intervention approaches.

All findings are interpreted as indicators of feasibility and implementation processes rather than evidence of intervention effectiveness and extend growth mindset theory beyond traditional educational contexts into the complex domain of residential care settings. Whereas most growth mindset interventions have focused primarily on academic achievement [5], our study demonstrates the potential and challenges of applying these principles to daily functional-skill development. The staff naturally adopted growth mindset language emphasizing effort, strategy development, and capacity for change [3]. However, the implementation gap between motivational messaging and structured functional-skill development suggests that residential care environments require specialized, context-specific intervention protocols.

### 4.1. Staff as Agents of Change

These findings provide preliminary support for the potential of residential care staff to be effective agents of positive change. High relationship-building capabilities align with extensive research emphasizing the critical importance of positive staff–youth relationships in determining residential care [37,38,39]. The enduring positive relationships alumnae described suggest these connections can be lasting protective factors that support successful community reintegration and long-term resilience.

Nevertheless, significant training needs emerged, particularly in functional-skill development methodologies and behavioral-regulation strategies. The observation that staff “can’t just enter and learn-as-you-go” reflects broader systemic concerns about staff preparation in complex care environments documented in the residential care literature [40]. The literature supports the effectiveness of structured, intensive training programs for paraprofessional staff [36] emphasizing the need for practice-based, experiential approaches rather than purely didactic educational models. Our feasibility findings corroborate these implementation considerations and informed the training component design.

### 4.2. Functional-Cognitive Models as Implementation Framework

The integration of functional-cognitive approaches with growth mindset [3,10] addresses a critical implementation gap identified in this study: the discrepancy between the staff’s growth mindset language and their difficulty translating these beliefs into concrete, functionally minded interventions that incorporate the adolescents’ personal goals. The CO-OP model’s emphasis on client-chosen goals, guided discovery, and explicit strategy development [19,20] provides a practical framework for operationalizing abstract growth-mindset principles into structured, functional interventions. The collaborative goal-setting processes described in the qualitative findings align closely with the CO-OP’s client-centered approach while incorporating the growth mindset’s emphasis on effort and strategy development rather than fixed-ability assumptions.

The multicontext approach’s systematic focus on skill transfer and generalization across diverse contexts [21,22] directly addresses another key implementation challenge: preparing youth for independent functioning beyond the residential facility. Within the pilot phase, transfer was addressed through staff-guided reflection linking in-facility functional goals to anticipated post-discharge or community-based situations.

The feedback limitations identified in this study, specifically the emphasis on outcome evaluation rather than on process and strategy development highlight the critical need for more sophisticated reflective practices consistent with functional-cognitive intervention approaches that promote metacognitive awareness and cross-contextual skill application. Our study provides a conceptual bridge between motivational and functional-cognitive frameworks, highlighting the role of occupational engagement in cultivating adaptive mindsets.

From a theoretical perspective, the integration of growth mindset, functional-cognitive approaches, and Self-Determination Theory can be understood as addressing complementary levels of the autonomy development process. Growth mindset primarily operates at the level of beliefs and motivation, shaping adolescents’ interpretations of challenge, effort, and failure, and influencing their willingness to engage in learning and strategy exploration. Functional-cognitive approaches, such as CO-OP and the multicontext approach, translate this motivational orientation into action by providing structured mechanisms for goal setting, strategy use, reflection, and transfer across everyday contexts. SDT offers an overarching motivational framework, clarifying how autonomy-supportive conditions, including choice, competence feedback, and relational safety, enable these processes to be internalized and sustained. Together, these perspectives suggest that motivation alone is insufficient in highly structured residential care settings unless it is paired with explicit scaffolding of metacognitive and functional processes that support autonomous performance [19,21,22,23].

Recent empirical studies conducted in residential care contexts further underscore the distinct characteristics of autonomy development among institutionalized adolescents [40]. A comparative study examining readiness for independent living among youth in residential childcare found that care-experienced youth reported relatively strong self-care and daily living skills yet demonstrated lower levels of community-based autonomy and independent functioning compared to peers without residential care experience [40]. These findings suggest that institutional environments may support certain structured life skills while simultaneously limiting opportunities for autonomous decision-making and transfer to less structured community contexts. Similarly, Oliveira [41] reported that adolescents and young adults living in institutional settings exhibited reduced levels of autonomy compared to non-institutionalized peers, particularly in domains related to self-determination and independent action. Together, these studies highlight that autonomy development in residential care is shaped by the structural and relational features of institutional environments, reinforcing the need to examine functional autonomy within its specific ecological context rather than extrapolating from school-based or clinical populations.

Rather than interpreting these findings as evidence of intervention effects, they are best understood through a theoretical lens that clarifies the mechanisms and contextual constraints involved. The findings are interpreted through a proposed integrative framework in which growth mindset is understood as a motivational precondition for engaging in metacognitive and functional strategy use, rather than as an outcome. This theoretical contribution helps explain the observed gap between strong relational support and limited opportunities for structured autonomy development in residential care contexts. These interpretations should be considered in light of the study’s limitations.

### 4.3. Organizational and Implementation Considerations

This study identified organizational factors that significantly influence intervention success. High staff turnover rates reflect broader systemic issues in residential care employment that must be addressed for sustainable implementation. Scheduling conflicts between educational staff and professional support teams highlight the need for comprehensive organizational commitment and adequate resource allocation for successful program integration. The intervention managed the structure–autonomy tradeoff by structuring the reflective and cognitive process while preserving youth choice and decision-making. These issues demonstrate the critical need for flexible implementation models that can adapt to crisis conditions while maintaining core intervention fidelity and therapeutic effectiveness.

### 4.4. Clinical and Practice Implications

This study provides evidence-based recommendations for enhancing residential care practice. Training programs should systematically build upon the existing staff’s relationship-building strengths while providing structured tools, ongoing supervision, and practice opportunities for functional-skill interventions. Integrating sensory-regulation strategies appears essential, given the at-risk adolescent population’s complex developmental and psychological needs.

The protocol structure requires significant adaptation to organizational realities, including flexible scheduling accommodations, small group training formats that promote peer learning, and comprehensive staff integration from program initiation through ongoing implementation. The emphasis on connecting immediate, achievable goals with longer-term aspirations appears crucial for sustained youth engagement and meaningful progress toward independence. It is important to note that the findings of this pilot study should be interpreted as identifying implementation needs, informing theoretical propositions, and guiding protocol refinement, rather than as evidence of intervention effects.

### 4.5. Limitations and Future Directions

Several methodological limitations affected the interpretation and generalizability of these findings. As a pilot, single-center study with small sample sizes and limited youth participation, the generalizability of the findings is constrained, particularly to male residential facilities and to different cultural contexts.

These data provide valuable contextual insights but do not allow for generalization or stable pattern identification. Rather, they serve as preliminary indicators that complement staff and alumnae perspectives and highlight areas for deeper examination in future studies with larger samples. The findings should therefore be interpreted with caution.

Further, the interrupted protocol implementation prevented a complete outcome evaluation, restricting conclusions to a feasibility assessment rather than determining intervention efficacy. The intervention interpretations should be considered in light of the study’s limitations, including the small pilot sample (n = 5), absence of a control condition, and focus on feasibility rather than effectiveness outcomes. 

Also, participation from the boys’ facility could not be organized due to competing operational priorities in the facility during the pilot period. These constraints highlight the importance of assessing organizational readiness and resource availability when considering future scale-up.

Due to the exploratory nature of the study and the limited sample size, advanced psychometric analyses of the Growth Mindset Facilitation Self-Efficacy questionnaire were not conducted. Further research with larger samples is needed to establish the factorial structure and additional validity evidence for this instrument. In addition, the use of self-report measures to assess staff competencies may have introduced social desirability bias; however, convergence with qualitative findings provided some support for the validity of the reported capabilities. The use of objective performance measures, such as the Canadian Occupational Performance Measure (COPM) [42], is often recommended. However, their application should be carefully considered, as the assessment process itself may impose additional cognitive or attentional demands on individuals with compromised attention span.

Limited current resident participation prevented adequate assessment of youth perspectives on the intervention’s acceptability and perceived effectiveness. Post hoc protocol modifications, although necessary for practical implementation, compromised systematic evaluation but provided valuable insights for future implementation planning.

Future research should examine intervention effectiveness through controlled evaluation designs with larger, more diverse samples and extended follow-up periods to assess sustained impact. Comparative studies across different facility types, organizational contexts, and cultural settings would significantly enhance understanding of the intervention’s generalizability and necessary adaptations. Investigating optimal training intensity, supervision models, and organizational support structures would inform evidence-based scaled implementation approaches. Longitudinal studies tracking youths’ outcomes after they leave the facility would provide crucial evidence on the success of community reintegration and the long-term impact of the intervention.

Technology-assisted delivery and monitoring systems warrant exploration as potential solutions to address logistical implementation challenges while maintaining intervention quality and fidelity. Additionally, cost-effectiveness analyses would support policy decisions on resource allocation and program sustainability, particularly relevant for public welfare systems facing budget constraints and competing priorities for at-risk youth services.

## 5. Conclusions

This mixed-methods feasibility study demonstrates that residential care staff may possess foundational capabilities for promoting growth mindset principles but require intensive, practice-based training to translate these relational strengths into structured, functional interventions effectively.

Although successful implementation requires substantial adaptation to the unique realities and constraints of residential care environments, the intervention protocol shows promise as a theoretically grounded bridge between abstract growth-mindset principles and practical daily-life skill development. These findings indicate that the integrated approach is feasible and acceptable within residential care settings while warranting further evaluation.

The study contributes preliminary evidence to the limited literature on the functional applications of growth mindset theory in residential care settings, demonstrating both the significant potential and substantial challenges of this therapeutic approach. Nevertheless, the findings should be interpreted as feasibility and acceptability indicators that inform future intervention development and evaluation of its effectiveness.

The novel integration of growth mindset theory with functional-cognitive intervention models represents a promising approach that warrants continued development and systematic empirical investigation. Furthermore, the protocol developed in this study may serve as a valuable framework for adaptation and implementation in similar residential care contexts, contributing to the global knowledge base for evidence-based youth residential care interventions.

## Figures and Tables

**Table 1:** Participant characteristics across groups.

Participant ID	Group	Gender	Role
S-1	Staff	Female	Educational counselor
S-2	Staff	Female	Social worker
S-3	Staff	Female	Educational counselor
S-4	Staff	Male	Educational counselor
S-5	Staff	Female	Program coordinator
S-6	Staff	Female	Educational counselor
S-7	Staff	Female	Educational counselor
S-8	Staff	Female	Educational counselor
S-9	Staff	Female	Facility director
Y-1	Youth	Female	Current resident
Y-2	Youth	Female	Current resident
Y-3	Youth	Female	Current resident
Y-4	Youth	Female	Current resident
A-1	Alumni	Female	Former resident
A-2	Alumni	Female	Former resident
A-3	Alumni	Female	Former resident

**Table 2:** Hierarchical Organization of Themes and Subthemes.

Category	Theme	Subtheme
**I. Facilitating factors**	Growth mindset integration and therapeutic language	Growth-mindset discourse reinforcing change capacity Competence affirmation through positive feedback Peer modeling and reinforcement of mindset language
	2. Collaborative goal-setting and youth agency	Alignment of goals with personal motivation Emphasis on locus of control and choice Staff-initiated activities promoting mastery experiences
	3. Environmental structure and regulation	Routine as regulatory mechanism Consistent boundaries and behavioral expectations Predictability reducing uncertainty and anxiety
	4. Relationship-based engagement	Therapeutic alliance between staff and youth Nonjudgmental stance as precondition for change Relational motivation and fear of disappointing staff Peer support networks and mutual encouragement
**II. Implementation challenges and systemic concerns**	5. Feedback practices and reflective learning	Outcome-focused versus process-focused feedback Limited metacognitive strategy development Insufficient reflective dialogue
	6. Transition to independent living	Functional skill gaps in community contexts Difficulty navigating external systems and services Need for graduated separation and ongoing support Post-discharge adjustment challenges
	7. Staff training and professional development	Preparedness gaps among newer staff Need for trauma-informed supervision and mentoring Importance of individualized, history-informed approaches Development of shared organizational discourse

## Data Availability

The original contributions presented in this study are included in the article/Supplementary Material. Further inquiries can be directed to the corresponding author.

## Supplementary Materials

The following supporting information can be downloaded at: https://www.mdpi.com/article/10.3390/children13010148/s1, File S1: Focus Group Interview Guide.

## Abbreviations

The following abbreviations are used in this manuscript:

CO-OP	The cognitive orientation to daily occupational performance
YCA	The Youth Custody Authority
SDT	Self-determination theory
