# Supplementary material for: Integrating Growth Mindset with Functional-Cognitive Approaches: A Mixed-Methods Feasibility and Acceptability Study in Youth Residential Care
Source: Children (Basel). 2026 Jan 20;13(1):148. doi: 10.3390/children13010148 (PMC12839644; doi:10.3390/children13010148)
Supplement: Supplementary file 1 [file children-13-00148-s001.zip › children-4085643-supplementary.pdf]

Focus Group Interview Guide

Questions to be asked during the focus groups

A. Focus Group for Staff Working in the Youth Custody Authority (YCA)**:**

1. What does your daily routine look like? What do you enjoy most during the day? What is most difficult?
2. In your opinion, what most helps within the YCA to enable residents to participate independently/positively or productively in daily life?
3. In your opinion, what is currently missing in the YCA that could enable residents to integrate optimally into daily life outside the facility?
4. What is forbidden/permitted to tell residents? And what is the reason for this?
5. What questions would you like to ask your graduates?
6. And what would you like us to ask the adolescents themselves?
7. If you could design a training program for new staff members, what content would you include?
8. What is important for a new staff member to know about the facility? Tips? Advice?
9. Brief explanation of what growth mindset is: what in this context connects to your daily work and what connects less? And why?

B. Focus Group for graduates of YCA:

1. What is your daily routine? Difficulty / enjoyment / frequency
2. What are you doing today?
3. What helped you most to get to where you are today?
4. Who helped you most during the time you were in the YCA, and describe this?
5. What helped you least during the time you were in the YCA, and describe this?
6. Did your peer group/friends help you? In what ways did they help and in what ways did they not?
7. What would you do differently if you could go back now to the time when you were in the YCA?
8. What has changed in your life since you left the protection facility?
9. Brief explanation of what growth mindset is: what about it is similar to things you experienced in the YCA and what is not similar?

C. Focus Group for adolescents currently in the YCA**:**

1. Describe your daily routine to us. What do you like most about your daily routine? What do you enjoy doing most during the day? What is most difficult?
2. What makes you feel good/less good about the place where you are?
3. What would you say/help/advise someone new who comes to the facility?
4. What would you most want to happen when you finish your time here?
5. A new counselor arrives here, what should they know? What would you advise them that would help them most with you?
6. In which things/activities can you decide for yourselves, and in which can you not?
7. Explanation of what growth mindset is: do you connect with this? What about it is similar to things you experience here and what is not similar?
